# A differential interplay between the expression of Th1/Th2/Treg related cytokine genes in *Teladorsagia circumcincta *infected DRB1*1101 carrier lambs

**DOI:** 10.1186/1297-9716-42-45

**Published:** 2011-03-08

**Authors:** Musa Hassan, James P Hanrahan, Barbara Good, Grace Mulcahy, Torres Sweeney

**Affiliations:** 1 School of Agriculture, Food Science, and Veterinary Medicine, University College Dublin, Dublin 4, Dublin, Ireland; 2 Teagasc Animal Production Research Centre, Athenry, Co. Galway, Ireland

## Abstract

Substantial debate exists on whether the immune response between sheep resistant and susceptible to gastrointestinal nematodes can be differentiated into a Th1 and Th2 phenotype. The present study addresses the hypothesis that variation in resistance to *Teladorsagia circumcincta *between DRB1*1101 (associated with reduced faecal egg count and worm burden) carriers and non-carriers is due to a differential interplay in the expression of Th1/Th2 and regulatory T (Treg) related cytokine genes. Lambs from each genotype were either slaughtered at day 0 (un-infected control) or infected with 3 × 10^4 ^*Teladorsagia circumcincta *L3 and slaughtered at 3, 7, 21, and 35 days later. Lambs carrying the DRB1*1101 allele had a significantly lower worm burden (*P < 0.05*) compared to the non-carriers. Abomasal mucosal cytokine gene expression was evaluated by quantitative real-time PCR and comparison made for time and genotype effects. The response generated varied through the course of infection and was affected by genotype. DRB1*1101 carriers had an up-regulated expression of the Th1-related cytokine genes (IL-1β, TNFα, and IFN-γ) at day 3, but this was replaced by an up-regulated expression of Th2-related cytokine genes (IL-10 and IL-13) and Treg-related cytokine genes (IL-2RA-CD25, TGFα, TGFβ, Arg2, MIF and FOXP3) by day 7. Conversely, in the non-carriers these changes in gene expression were delayed until days 7 and 21 post infection (pi), respectively. It is concluded that resistance to *Teladorsagia circumcincta *in animals carrying the DRB1*1101 allele is influenced by an earlier interplay between Th1, Th2 and T regulatory immune response genes.

## Introduction

The abomasal parasite, *Teladorsagia circumcincta *(*T. circumcincta*) is a major impediment to sheep production in temperate and subtropical climates [1] both in terms of animal welfare and economic loss [2,3]. While anthelmintics can be used to control gastrointestinal nematodes, the increased incidence of drug resistance [4-6] and demands for meat products free of chemical residues [7] have led to greater interest in alternative parasite control strategies.

While both cellular and humoral immune responses contribute to protective immunity against gastrointestinal nematodes, the dichotomy of protective immunity against gastrointestinal nematodes remains unclear in sheep. Susceptibility or resistance to gastrointestinal nematodes in sheep has often been attributed to a CD4^+ ^T-helper type 1 (Th1) or CD4^+ ^T-helper type 2 (Th2) immune responses. Indeed depletion of CD4^+ ^T helper cells abrogated resistance to *Haemonchus contortus *(*H. contortus*) [8]. Similarly, expression of Th2 related cytokines was observed in mesenteric lymph [9], abomasal lymph nodes [10-12], abomasal mucosa [13], and blood [14] of sheep infected with gastrointestinal nematodes. However, there is evidence for the concurrent expression of Th1 and Th2 genes [9,15] and more recent evidence for the involvement of immunoregulatory genes [12,13,16] in animals undergoing nematode challenge. This evidence puts into question the existence of a clear Th1/Th2 dichotomy. Additionally, immune regulatory cells such as fork-head box-P3^+ ^(FOXP3^+^) [17] and CD25^+ ^T lymphocytes [18] have been observed in sheep undergoing *T. circumcincta *infection. Therefore, susceptibility to *T. circumcincta *may not be due to a Th1/Th2 balance but rather a failure of immunoregulatory (Treg) responses that terminate inappropriate inflammatory responses, whether Th1 or Th2, while allowing the development of essential immune responses.

In the current study a group of DRB1*1101 carrier and non-carrier lambs, experimentally infected with *T. circumincta *was used in a time-course mucosal gene expression assay to characterise the chronological changes in Th1-, Th2- and Treg-related cytokine gene expression. It has previously been shown that lambs carrying the Ovar- DRB1*1101 allele are more resistant to *T. cirumcincta *than non-carriers of this allele, as measured by worm burden [19].

## Materials and methods

### Animals and experimental design

Six unrelated sires heterozygous for the DRB1*1101 allele were mated with ewes in single sire groups to generate progeny carrying or lacking the DRB1*1101 allele. This allele was initially named G2 [20,21] and DRB1*0203 [22]. However due to the international standardisation of the nomenclature of the MHC alleles, this allele is now known as the DRB1*1101 [23]. All lambs were genotyped at the *Ovar-DRB1 *locus, by short tandem repeat (STR) analysis and confirmed by direct sequencing of genomic DNA, as previously described [22,24]. It was intended that lambs used in the study would be twin pairs where one sibling was a carrier of the DRB1*1101 allele and the other sibling a non-carrier. Within the constraints of birth date, birth type and lamb survival, 18 suitable twin pairs were identified. These were randomly assigned to one of five slaughter dates (days 0, 3, 7, 21, and 35 of the experiment) subject to the constraints that there were a maximum of four pairs per slaughter date. To maintain numerical balance in the design, we utilised a twin pair where both siblings were carriers on day 3 and another pair where both siblings were non carriers on day 21. The lambs were born indoors at the Teagasc Animal Production Research Centre, and were put to pasture until they were between 4 and 5 weeks of age when they were moved back indoors with their dams. All lambs were weaned 1 week later and faecal sampled to determine faecal egg count 3 weeks after weaning (80% of the lambs had a FEC < 150 and the maximum FEC observed was 250) and treated with Oramec (Merial Animal Health Ltd, Essex, UK) as per the manufacturer's instructions. The lambs were moved to Lyons Research Farm, University College Dublin, at about 10 weeks of age, housed in a nematode free environment and given 2 weeks to acclimatise prior to the start of the experiment. The lambs were free of nematode infection at the start of the experiment, based on FEC measurements on three consecutive days. All procedures described in this experiment were conducted under experimental licence from the Irish Department of Health in accordance with the Cruelty to Animals Act 1876 and the European Communities (Amendments of the Cruelty to Animals Act, 1976) Regulations, 1994. The lambs received granulated feed and water *ad libitum*. At about 12 weeks of age, eight lambs were slaughtered (day 0; controls) and the remaining lambs each received a single oral dose of 3 × 10^4 ^*T. circumcincta *L3. The subsequent slaughter dates were chosen based on the developmental stages of the parasite in the sheep abomasum; day 3 (L3 stage), day 7 (L4 stage) and days 21 and 35 (egg laying stage).

### Tissue collection

The abomasum was removed at slaughter and rinsed in saline. The abomasal mucosa was then removed by scraping with a blunt blade. The abomasal scrapings from these lambs and the unchallenged lambs were preserved in RNAlater^® ^(Applied Biosystems, Warrington, UK) and stored at -20°C until use.

### RNA extraction

RNA was extracted from the stored abomasal scrapings using TRI Reagent^® ^(Bio-Sciences Ltd, Dublin, Ireland) as per the manufacturer's recommendations. Samples were homogenised in 1 mL TRI Reagent^® ^using a Retsch^® ^tissuelyser (Qiagen, Crawley, UK) and 0.2 mL chloroform (Sigma Aldrich, Wicklow, Ireland) added for phase separation. The aqueous phase was transferred to a fresh eppendorf tube (Eppendorf, Hamburg, Germany) and mixed with 0.5 mL isopropanol. This was then centrifuged at 10 000 × *g *for 15 min at 4°C to collect the RNA pellet. The pellet was then washed in 75% ethanol and dissolved in RNAse free water.

RNA clean-up was performed using the RNeasy^® ^mini kit (Qiagen) as per the manufacturers' recommendations. RNA was quantified by spectrophotometric measurements at 260 nm and purity was assessed by determining the OD ratio 260/280 nm. RNA integrity was assessed using Agilent^® ^2100 bionalyzer (Agilent Technologies, Cork, Ireland). Only samples with RNA integrity number (RIN) above eight were used in the gene expression assay.

### Reverse transcription of mRNA into cDNA

Total RNA was transcribed into cDNA using RevertAid™ H minus First strand cDNA synthesis kit (Fermentas, St. Leon-Rot, Germany) and oligo(dT)_18_. Each 40 μL reaction was set up on ice and consisted of 2 μg RNA, 1 μg oligo(dT)_18 _primer, 1X reaction buffer, 20 units Ribolock™ Ribonuclease inhibitor, 1 mM dNTP mix, 400 units RevertAid™ H minus M-MuLV RT and made up to 40 μL using DEPC treated water. Subsequent to incubation at 42°C for 1 h, the enzyme was deactivated at 70°C for 5 min and the cDNA chilled on ice. The cDNA was then made up to 200 μL using RNAse free water and stored at -20°C. A 1:2 serial dilution made from a pool of all the cDNA samples was also stored at -20°C.

### Quantitative Real-Time PCR assay

Quantitative real time PCR (qPCR) was used to determine transcript abundances for the 19 genes and 5 reference genes described in Table 1. To minimise potential variations, samples from all the time points were set up on the same plate for each gene. Further, the efficiency of the qPCR was determined for each gene [25]. qPCR was performed on ABI Prism 7500 Fast sequence detection system (Applied Biosystems). Each 20 μL reaction contained 1 × Fast SYBR green Master Mix (Applied Biosystems), 5 mM of each primer and a constant amount of cDNA, corresponding to 10 ng of transcribed RNA for each sample. For each gene, an aliquot of the pooled cDNA sample was set up along with the test samples and used to calculate the PCR efficiency [25].

**Table 1:** A list of genes quantified by qPCR in the abomasal mucosa of DRB1*1101 carrier and non-carrier lambs following infection with 3 × 10^4 ^*T. circumcincta *L3.

Functional grouping	Gene names
Cytokine-cytokine receptor signalling	Th1 (IL1β, IL12, IFNγ, TNFα,)
	Th2 (IL4, IL10, IL13) immunoregulatory (IL2RA-D25, FOXP3, Arg2, MIF, TGFα, TGFβ)
Extracellular matrix	TFF2, TFF3, Ovar-Gal14, sITLN2, sMCP-1, GATA-3
Reference genes	GAPDH, ATPase, RPLP0, ACTB, 18s rRNA

The oligonucleotide primers used to assay these genes have been previously published [12,13,26]. To normalise the expression data, a normalisation factor was derived from the five reference genes using Genorm^® ^[27]; a combination of the ATPase and RPLP0 genes provided the best normalisation factor.

### Data analysis

The normalisation factor was used to determine relative gene expression values at each time point [25]. A *t*-test was then used to evaluate the difference between the genetic groups at each time point post infection (pi) at 5% level of significance.

## Results

### Worm numbers

Detailed parasitology data obtained post-mortem from the abomasum contents and digest are presented in [19]. Significantly (*P < 0.05*) lower numbers of *T. circumcincta *were recovered from the abomasum of DRB1*1101 carriers on days 21 and 35 (day 3 geometric mean, 8 919; day 7 geometric mean, 15 383, day 21 geometric mean, 12 654, day 35 geometric mean 2 228) compared to the non-carriers (day 3 geometric mean 7 149; day 7 geometric mean 19 062, day 21 geometric mean 20 836, day 35 geometric mean 8 333).

### Relative cytokine expression

**Figure 1:**
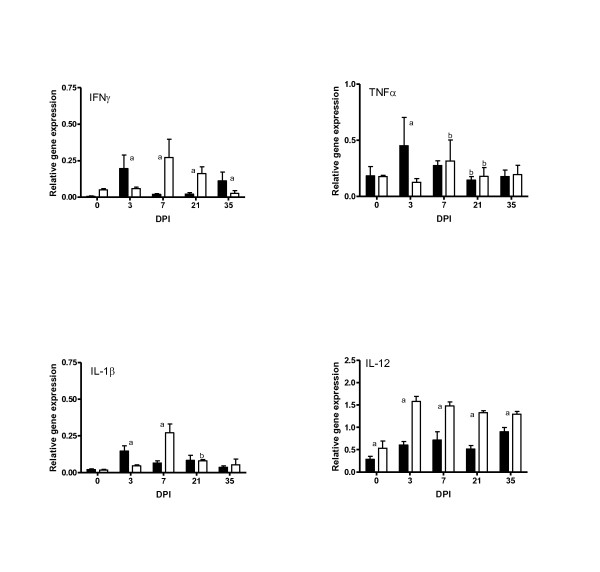
Relative expression of Th1 cytokines in the abomasal mucosa of DRB1*1101 carrier (black bars) and non-carrier (white bars) lambs following infection with 3 × 10^4 ^*T. circumcincta *L3. (a = significant differences between genetic groups; b = significant differences with the preceding time point).

The expression levels of the Th1-related cytokine genes (IFNγ, TNFα, IL-1β, and IL-12) are presented in Figure 1. Following infection, a significant up-regulation of IFNγ, TNFα and IL-1β was observed on day 3 (*P < 0.05*) in the DRB1*1101 carriers compared to the non-carriers, followed with significant down-regulation by day 7. Up-regulation of IL-1β, IFNγ and TNFα was not observed in the non-carriers until day 7 pi followed by a significant down-regulation by day 21.

The expression pattern of IL-12 was different to the other Th1 cytokines examined. IL-12 expression was up-regulated by day 3 in all genotypes and remained elevated for the duration of the experiment. The non-carrier lambs had significantly (*P < 0.05*) higher level of IL-12 than the carrier lambs.

**Figure 2:**
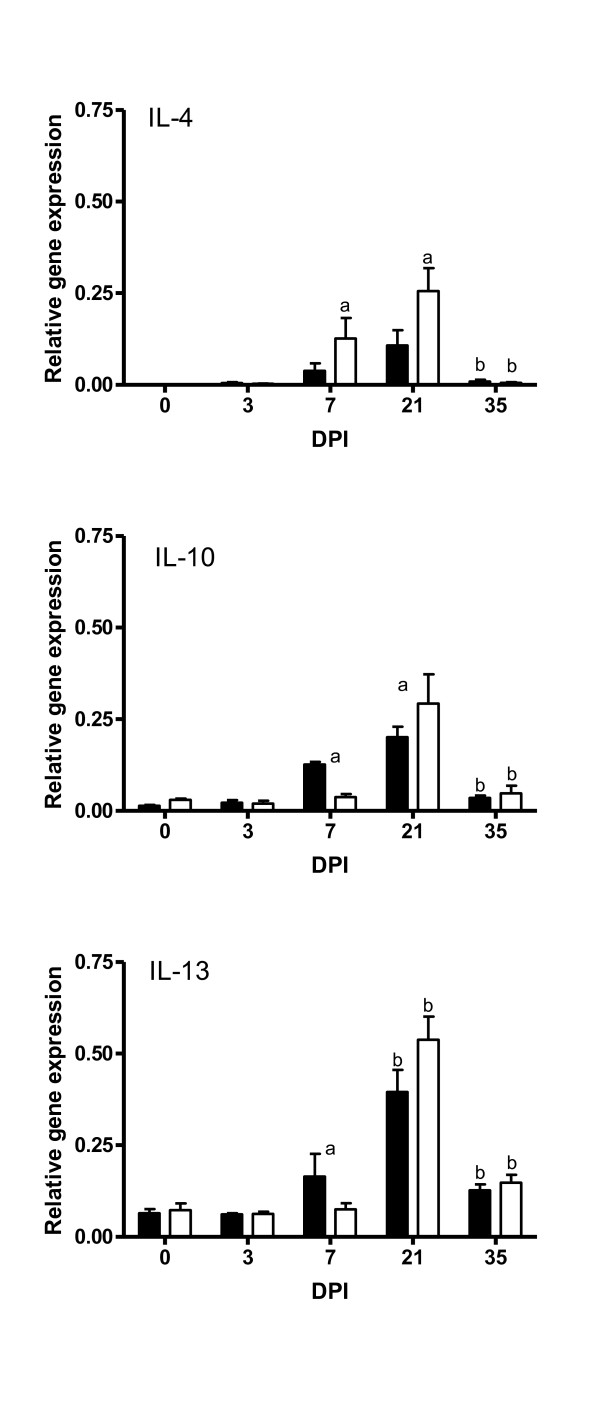
Relative expression of Th2 related cytokine genes in the abomasal mucosa of DRB1*1101 carrier (black bars) and non-carrier (white bars) lambs following infection with 3 × 10^4 ^*T. circumcincta *L3. (a = significant differences between genetic groups; b = significant differences with the preceding time point).

The expression levels of the Th2-associated cytokine genes (IL-4, IL-10, and IL-13) are presented in Figure 2. There was a significant up-regulation of IL-10 and IL-13 by day 7 in the carrier lambs and by day 21 in the non-carrier lambs. The up-regulation of these cytokines was followed by a significant down-regulation by day 35 in both groups. On the other hand, there was a significant up-regulation of IL-4 from day 7 followed by a down-regulation on day 35 in both groups.

**Figure 3:**
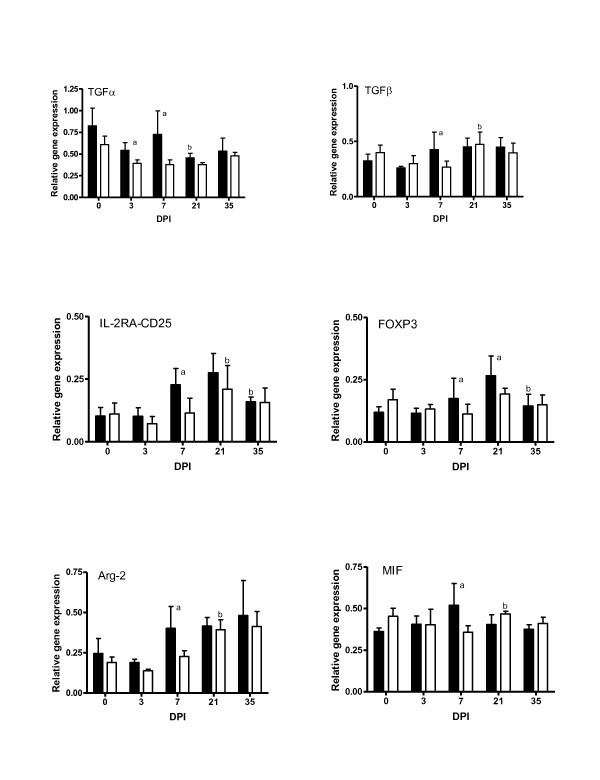
Relative expression of immunoregulatory related cytokine genes in the DRB1*1101 carrier (black bars) and non-carrier (white bars) lambs following infection with 3 × 10^4 ^*T. circumcinta *L3. (a = significant differences between genetic groups; b = significant differences with the preceding time point).

The expression levels of immunoregulatory-related genes (TGFα, TGFβ, IL-2RA-CD25, FOXP3, Arg2, and MIF) are presented in Figure 3. Significant (*P < 0.05*) increases in all the immunoregulatory cytokine genes assayed were observed on day 7 in the carriers compared to the non-carriers. However, the transcript levels for TGFβ, IL-2RA/CD25, FOXP3, Arg2, and MIF increased significantly (*P < 0.05*) on day 21 in the non-carriers to reach levels similar to those observed in the carrier lambs on the same day.

### Extracellular matrix and mucosal integrity genes

**Figure 4:**
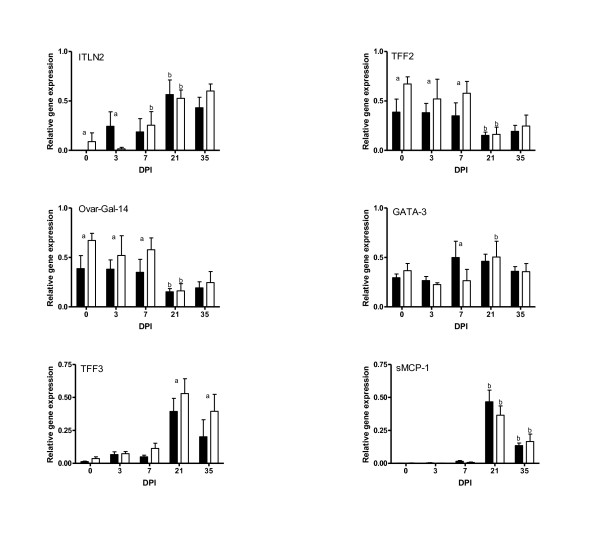
Relative expression of extracellular and mucosal integrity genes in the DRB1*1101 carrier (black bars) and non-carrier (white bars) lambs following infection with 3 × 10^4 ^*T. circumcinta *L3. (a = significant differences between genetic groups; b = significant differences with the preceding time point).

The expression of mucosal integrity genes are presented in Figure 4. Before infection, the non-carriers had significantly (*P < 0.05*) elevated levels of ITLN-2, TFF2, and Ovar-Gal14 compared to the carriers. Following infection, the carrier lambs had significantly (*P < 0.05*) higher levels of ITNL2 on day 3 and GATA3 on day 7 compared to the non-carriers. The expression of TFF2 and Ovar-Gal14 was elevated until day 7 and was then down regulated on day 21 in both genetic groups. In contrast, there was an increased expression of TFF3, and sMCP-1 from day 21 in both groups.

## Discussion

The outcome of infection is dependent on the balance between both the host survival and parasite virulence strategies [28,29]. In experimental nematode infections, susceptibility is thought to be associated with a Th1 immune response [12]. However, a prolonged inflammatory response would not be desirable to the host. On the other hand, resistant animals elicit a strong Th2 response, which is potentially hostile to the fitness strategy of nematodes [13]. Thus, the parasite may attempt to constrain or modulate the Th2 response in order to survive. It is not known if the parasites deregulate the Th2 response by down-regulating specific cytokines and inducing development of regulatory T cells, or if they down-regulate the Th2 response in favour of a Th1 response. In the present study, there was a rapid shift from a Th1 immune response characterised by the expression of IFNγ and IL-1β genes to a regulated Th2 immune response characterised by the expression of IL-13, IL-10, TGFβ, and FOXP3 genes by seven days post infection (dpi) in the carrier lambs. However, in the non-carrier lambs, the switch from the predominantly Th1 immune response was delayed until 21 dpi. Interestingly, the timing of the Th1/Th2/Treg immune switch coincided with the decline in worm burden in both group of lambs.

Increased expression of IL-10 and IL-13 was observed on day 7 in the DRB1*1101 carriers. Conversely, the up-regulation of these cytokines was delayed until day 21 in the non-carriers. Similar anti-inflammatory responses have been observed in sheep infected with *H. contortus *[11,30], *T. colubriformis *[31] and *T. circumcincta *[12], and in cattle infected with *Ostertagia ostertagi *[32]. IL-13 is known to induce Th2 cell responses and promote the production of IgE [33]. IgE has been associated with increased resistance to *T. circumcincta *[34,35] and the IgE levels were up-regulated in the mucosa of these lambs on day 7 pi [19].

The up-regulation of TGFβ, IL-2RA-CD25, FOXP3, Arg-2 and MIF on day 7 in the carrier lambs in the present study may reflect the role of these genes in immune regulation. Previously, FOXP3^+ ^T cells [17], CD25^+ ^T cells [18] and an up-regulation of IL-10 and TGFβ genes [12] have been observed in of sheep infected with *T. circumcincta*. Treg cells play a significant role in suppressing excessive immune response that may be deleterious to the host [36,37]. The cost of mounting an effective immune response against gastrointestinal nematodes has been extensively reviewed [38-40]. It is therefore argued, that in the interest of the host, the immune response be attenuated as soon as it has achieved its objective to enable the diversion of nutrients to other cellular functions, including growth. The interleukin-2 receptor alpha (IL-2RA) and FOXP3 are key regulatory genes for the development and function of cells with immunoregulatory functions [41]. Different types of Treg cells in humans and mice including CD4^+ ^have been shown to express high levels of IL-2RA and such cells are a major proportion of the population of Treg cells [36]. IL-2RA/CD25^+ ^knock-out mice have been shown to develop autoimmune disease [42], suggesting that this gene is needed for immune regulation. Arg-2 and MIF are considered markers of alternatively activated macrophages, markers of immune regulation [43,44]. Arg-2 is known to abrogate nitric oxide (NO) production, resulting in the inhibition of IFNγ-mediated apoptosis [45] a feature of susceptibility to gastrointestinal nematodes [16]. On the other hand, MIF is a critical mediator in innate and acquired immunity, exhibiting inflammatory functions [46] and anti-apoptotic activity [47]. MIF is produced by alternatively activated macrophages [48] and sustains macrophage survival and function by suppressing activation-induced apoptosis [49]. MIF is also known to influence IL-5-dependent tissue eosinphilia induced by *Schistosoma mansoni *infection in mice [50], and also plays a role in the control of worm fecundity in mice infected with *Schistosoma japonicum *[51]. It is interesting in this context that the expression of these molecules preceded a reduction in worm numbers in the carrier lambs. Nematodes have been reported to produce mouse homologues of this protein, and perhaps use it to modulate the host immune response, by inducing of alternative activation of host's macrophages [52]. The common denominator in these two molecules is their anti-apoptotic activity and immune regulation, processes associated with resistance to gastrointestinal nematodes [53,54]. The increased expression of anti-apoptotic proteins, such as heat shock protein 70, and B-cell leukaemia/lymphoma 2 (Bcl2), in carrier lambs at the same time point while the non-carriers had increased expression of apoptosis related peroxiredoxin-2 (Hassan *et al*., The proteome of the abomasal mucosa of lambs infected with *Teladorsagia circumcincta *- an important role for apoptosis? Prepared Manuscript) indicates that the expression of immunoregulatory genes is not only important in suppressing excessive immune response, but also in regulating cell survival and viability.

The significant up-regulation of trefoil factor 3 (TFF3) in both genetic groups by day 21 pi is consistent with the suggestion that the viscosity of luminal contents may contribute to nematode localisation [13]. TFFs are thought to be important in protecting the mucosa from insults [55-58] and the production of TFF3 is up-regulated by IL-4 and IL-13 [59]. It is therefore, feasible that the observed up-regulation of TFF3 is a response to IL-4 and IL-13 production or mucosal damage following infection. The current study shows an up regulation of sheep interlectin-2 (sITLN-2) from day 3 in carrier lambs and from day 7 in the non-carriers relative to their corresponding day 0 controls. An up-regulation of sITLN-2 has been observed on day 10 in naive and day 3 in previously infected sheep following *T. circumcincta *challenge [26]. Interlectins are thought to play a role in altering the characteristics of mucus leading to parasite entrapment [60].

The increased expression of sMCP-1, a marker of mucosal mast cells [61,62], from day 21 in both genetic groups following *T. circumcincta *infection could be attributed to increased mast cell infiltration in the abomasal mucosa (as observed in these lambs at the same time point; [19]). Increased mast cell infiltration is a characteristic feature of ovine response to nematode infection [63-65]. GATA-3 is reported to be essential in the maintenance of Th2 cytokine production [66]. It is interesting in this context in that the up-regulation of GATA-3, on day 7, occurred at the same time point as the up-regulation of Th2 related cytokine genes.

The lack of a significant difference in some of the interleukins, such as IL-4, could be due to the tissue sampled rather than differential expression between the lambs. T cell - dendritic cell (DC) interactions in the lymph node is a key step in T cell priming [67]. Since most pathogens, including nematodes, enter the body through non-lymphoid tissue, tissue resident DCs depend on pathogen derived signals to mature and migrate to the local lymph nodes, where they present antigens to naïve T cells [68]. Additionally, T cell activation is dependent on eliciting an intracellular signal which is strong enough to irreversibly commit the cell to differentiate [69]. Consequently, the mucosa not being the recognised site of T cell-DC interactions may result in weak signals hence less T cell activation. Analyses of the abomasal lymph node may provide a better snap shot of the T cell specific immune response than the abomasal mucosae. However, the lymph node being the main site of T- dendritic cells interactions may skew the observed expression of cytokines since this will represent expressions induced by all pathogens resident in the host at the time of sampling. It is noteworthy that, except for a few cytokines, results obtained from independent assays done on lymph nodes draining the abomasal mucosa [12,18] or the abomasal mucosa [13] are comparable.

In general, effector immune molecules will not be immediately available following invasion by a novel pathogen [70]. A time delay between infection and immune initiation and a gradual build-up in immune efficacy is expected to occur following infection [71]. Therefore the sampling time can significantly influence the immune factors observed in infected animals. From the current study, it appears that the DRB1*1101 carrier lambs had a shorter time delay relative to the non-carrier lambs. By day 3, DRB1*1101 carrier lambs were clearly expressing pro-inflammatory cytokines IL-1β and IFNγ, whereas similar observations were not made until day 7 in the non-carriers. Interestingly, by 35 dpi, the cytokine levels in both genetic groups had returned to pre-infection levels. This can partly be attributed to down regulation of the cytokine production to limit the energy cost associated with a strong immune response [38,39]. Even though the cytokine environment is considered the key factor in determining naïve T cell differentiation [72,73], other factors including antigen dose, ligand alterations and antigen presenting cells can influence naïve T cell differentiation [74]. Consequently, the expression of MHC-II molecules on the APC may play an important role in initiating a faster T cell response. However, how the MHC-II alleles can lead to the faster immune response in resistant compared to the susceptible lambs in the current study is not clear since it has been reported that there seems to be no differences in antigen recognition amongst the various MHC-II alleles [75]. Nevertheless, a faster immune response has also been reported in resistant lambs compared to susceptible lambs infected with gastrointestinal nematodes [11,76]. Since there is a shorter time to respond to the infective larvae before it moves to abomasal mucosa (day 3 to 7, Barbara G, personal communication), it would appear that a faster T cell immune response in the carrier lambs, enables these lambs to control the parasite burden by reducing the number of larvae that do eventually develop into adults. It is indicative that a key feature of nematode resistance is the inhibition of larval development at the tissue-residing L4 stage [77,78]. A similar switch from a Th1 to a Th2 and T-regulatory immune response has also been observed in schistosomiasis [79,80]. A possible explanation for the lack of a clear Th1/Th2 phenotype in sheep is the expression of immunoregulatory genes. From these results, it can be concluded that variation in resistance to gastrointestinal nematodes is due to differential interplay in the expression of genes involved in Th1, Th2, and immunoregulatory responses.

## Competing interests

The authors declare that they have no competing interests.

## Authors' contributions

JP, TS and GM conceived of the study. MH, JP, TS, and BG designed the experiments. BG and MH collected samples. MH carried out the experiments and drafted the manuscript. All the authors have read and approved the manuscript.
